# A model predicting the 6-year all cause mortality of patients with advanced schistosomiasis after discharge: Derived from a large population-based cohort study

**DOI:** 10.1371/journal.pntd.0013134

**Published:** 2025-05-27

**Authors:** Lanyue Pan, Chunmei Wu, Ping Li, Jiaquan Huang, Yizhi Wu, Guo Li

**Affiliations:** 1 State Key Laboratory for Diagnosis and Treatment of Severe Zoonotic Infectious Diseases, Department and Institute of Infectious Disease, Tongji Hospital, Tongji Medical College, Huazhong University of Science and Technology, Wuhan, Hubei Province, China; 2 Department of Neurology, Tongji Hospital, Tongji Medical College, Huazhong University of Science and Technology, Wuhan, Hubei Province, China; Cyprus International University: Uluslararasi Kibris Universitesi, CYPRUS

## Abstract

**Background:**

Advanced schistosomiasis imposed a heavy economic burden on society and had a high rate of mortality and disability. However, methods for assessing its long-term prognosis were currently insufficient, and there was a lack of predictive tools to aid clinical decision-making and personalized follow-up plans for patients. We sought to determine risk factors associated with six-year all-cause mortality in advanced schistosomiasis, deriving and validating a six-year all-cause mortality prediction model through a retrospective cohort study based on a large population-based cohort.

**Methodology:**

We collected information from 4,136 patients with advanced schistosomiasis who were discharged between December 2014 and January 2015. After excluding 17 patients with the less common subtypes of colonic tumoroid proliferation and dwarfism, as well as 92 patients who were lost to follow-up or had incomplete information, data from 4,027 patients were included in the study. These patients were randomly assigned to the derivation cohort and the external validation cohort in a 7:3 ratio, with 1,400 patients randomly selected from the derivation cohort for internal validation. Sixteen candidate variables were collected: age, gender, nutritional status, splenectomy history, presence of other conditions (such as cardiovascular and digestive diseases), clinical classification, disease duration, ascites occurrence frequency, levels of serum total bilirubin (TBil), direct bilirubin (DBil), aspartate aminotransferase (AST), alanine aminotransferase (ALT), albumin (ALB), alkaline phosphatase (ALP), Hepatitis B surface antigen (HBsAg), and alpha-fetoprotein (AFP). High-risk factors associated with the 6-year mortality outcome were identified through univariate and multivariate Cox proportional hazards regression analyses. The predictive value of different models was evaluated and compared using the receiver operating characteristic (ROC) curves, Akaike information criterion (AIC), net reclassification improvement (NRI), C statistic, and integrated discrimination improvement (IDI).

**Findings:**

The derivation cohort comprised 2819 patients and we randomly selected 1400 cases from this cohort for internal validation. The external cohort consisted of 1208 patients. The mortality rate for three groups was around 27%-28%. We identified ten variables associated with increased risk of death, including age, course of disease, frequence of ascites, hepatitis B co-infection, and levels of DBil, ALT, AST, ALP, ALB, and AFP at baseline. Using these variables, we developed a ten-variable model and three simpler models. In the derivation cohort, the ten-variable model showed the highest C statistic (0.759; 95% CI, 0.739-0.778) and the lowest AIC (2834.2). ROC curves indicated an AUC of 0.759 for the ten-variable model, outperforming the simpler models. External validation also demonstrated superior performance of the ten-variable model with a higher C statistic (0.774; 95% CI, 0.749-0.797). This model consistently showed better results in ROC curves, IDI, continuous NRI, and categorical NRI analyses compared to the reduced models in external validation cohort.

**Conclusions:**

This study developed a multivariate model to predict the 6-year all-cause mortality rate in patients with advanced schistosomiasis, which demonstrated good performance. This convenient tool may potentially assist clinicians in formulating patient follow-up plans.

## Author summary

Existing methods for evaluating the long-term prognosis of advanced schistosomiasis were inadequate and needed improvement. This study aimed to identify risk factors associated with six-year all-cause mortality and to develop a predictive model using data from a large population-based cohort. We identified ten factors linked to an increased risk of death in patients with advanced schistosomiasis. Using these variables, we constructed a predictive model and validated its performance through various statistical measures, including ROC, AIC, NRI, and IDI, in both internal and external validation cohorts. The model that included all ten variables outperformed the reduced models. These findings highlight key indicators associated with six-year mortality in patients with advanced schistosomiasis, and the predictive model demonstrated strong performance through validation. This model may assist clinicians in making more informed decisions and developing personalized follow-up plans, potentially improving survival outcomes for patients with advanced schistosomiasis.

## Introduction

Schistosomiasis included three distinct phases based on clinical manifestation and progresses: acute, chronic and advanced period [1]. As a type of the neglected tropical diseases, it spreads across 78 countries and regions, resulting in a heavy economic burden [2–4]. As reported by the Global Burden of Disease Study 2021, the global burden of schistosomiasis was estimated at 856 thousand all-age disability-adjusted life-years (DALYs) [5], nearly a 50% decrease from the 1.9 million DALYs assessed in the Global Burden of Disease Study 2016 [6]. This remarkable result was attributed to a series of sustainable strategies, such as enhancements in water infrastructure, sanitation and hygiene (WASH), reducing intermediate host snails, preventive chemotherapy, especially control of infection sources [7–10]. Due to effective control measures, the prevalence of schistosomiasis among humans and livestock has markedly decreased, resulting in a corresponding decline in the incidence of acute schistosomiasis. For example, in China, the incidence of acute infections in 2012 significantly decreased compared to 2005, with a reduction of 97.7% [11]. After mass drug administration in Burkina Faso, Africa, the prevalence of heavy-intensity schistosomiasis infections among children aged 5–14 in 2023–2024 dropped to 0.9%, reflecting nearly a 90% reduction from the baseline prevalence [12]. Despite the decline in the burden of schistosomiasis, it has not yet reached the desired target. The disease continues to exert a substantial burden, particularly in some underdeveloped regions [13].

The Global Burden of Disease (GBD) study currently did not place significant emphasis on chronic and advanced schistosomiasis when assessing the disease burden. In fact, the global burden of schistosomiasis caused by chronic and advanced cases may be largely overlooked [14]. In recent years, the incidence of acute schistosomiasis has decreased sharply, with many regions approaching a near-zero incidence even in heavy-intensity areas [12,15–17]. However, the global burden of schistosomiasis remains substantial and the burden of disease has actually increased in high Socio-Demographic Index (SDI) regions and countries [18]. Considering the significant decrease in new cases alongside heavy burden of schistosimiasis, we speculated that chronic and advanced cases may contribute significantly to the overall disease burden.

In China, four clinical subtypes of advanced schistosomiasis were classified: ascites, megalosplenia, colonic tumoroid proliferation, and dwarfism. Ascites and megasplenia, the most common types, were usually associated with serious complications such as portal hypertension and gastroesophageal variceal bleeding, which can present as life-threatening emergencies. Due to the severity of advanced schistosomiasis, cases in China were treated and managed separately from chronic cases [19]. Unfortunately, the burden of advanced schistosomiasis remains underrecognized and significantly underestimated in many countries, despite its serious consequences. While numerous studies have focused on general chronic schistosomiasis, there was a notable lack of long-term survival research specifically addressing advanced schistosomiasis. Considering the adverse outcomes, it was imperative to implement more precise and personalized follow-up programs for patients with advanced schistosomiasis to improve their survival and overall well-being.

In fact, there were only a limited number of studies on prognostic models for advanced schistosomiasis. In previous studies, our group developed prognostic prediction models for advanced schistosomiasis using competing risk analysis, survival analysis, and machine learning techniques. Subsequently, similar studies have been conducted, which developed a 1-year prognostic prediction model for advanced schistosomiasis [20–23]. The follow-up periods in the above studies were relatively short, ranging from 1 to 2 years. Considering the prolonged course of advanced schistosomiasis, it is essential to conduct long-term studies to expand upon previous research and improve clinical decision-making and patient management. Taking advantages of independent management in China, we were able to collect highly valuable long-term follow-up data on advanced schistosomiasis, achieving extremely low loss rate.

In this study, we performed a large-sample, long-term follow-up retrospective cohort analysis, employing methodologies consistent with previous research [21]. We collected demographic, laboratory, and clinical survival outcome data from registered patients with advanced schistosomiasis in Hubei Province to derive and validate the 6-year mortality risk associated with advanced schistosomiasis and developed a predictive model for 6-year all-cause mortality. This endeavor aimed to provide in-depth insights into the long-term prognosis of these patients through large cohort data, to enhance the utilization of healthcare resources and improve patient survival rates.

## Materials and methods

### Ethics statement

We developed prediction models in line with the TRIPOD statement [24], adhering to ethical standards established by the 1964 Helsinki Declaration and its subsequent updates. To safeguard patient privacy, all data were anonymized and personal information was appropriately de-identified. The study received approval from the Ethics Committee of Tongji Medical College, Huazhong University of Science and Technology (Approval number: TJ-IRB202408047). As it involved minimal risk and no interventions, informed consent was waived, ensuring no adverse impact on subjects’ rights or welfare.

### Study population and model development

We developed both derivation and validation cohorts using a population-based database that included patients from Hubei Province, China, diagnosed with advanced schistosomiasis. The discharge period for patients ranged between September 2014 and January 2015, with follow-ups continuing until January 2021. Patients were advised to attend regular follow-up appointments, typically scheduled every three months during the initial two years and annually thereafter. For patients who missed their scheduled visits, follow-ups were conducted via telephone to gather treatment details and assess survival status. The primary endpoint was all-cause mortality over a six-year period post-discharge. Any deaths occurring after January 2021 were not considered events in our analysis.

We collected clinical data from 4,136 cases. After excluding 109 patients due to small sample sizes for certain disease subtypes (4 cases diagnosed with colonic tumoroid proliferation and 13 diagnosed with dwarfism) or loss to follow-up (n = 92), 4,027 patients were incorporated in the analysis. These patients were randomly assigned to a derivation cohort (n = 2,819) and an external validation cohort (n = 1,208) in 7:3 ratio. Additionally, 1,400 cases were randomly selected from the derivation cohort for internal validation. As in the previous study, 16 variables were considered for analysis: age, gender, nutritional status, splenectomy history, presence of other conditions (such as cardiovascular and digestive diseases), clinical classification, disease duration, ascites occurrence frequency, levels of serum total bilirubin (TBil), direct bilirubin (DBil), aspartate aminotransferase (AST), alanine aminotransferase (ALT), albumin (ALB), alkaline phosphatase (ALP), Hepatitis B surface antigen (HBsAg), and alpha-fetoprotein (AFP). The inclusion and exclusion criteria, diagnostic criteria for patients, variable selection, model derivation, and validation of predictive capabilities have been detailed in our previous study. [21] Descriptive statistics were used to analyze the baseline characteristics of the derivation, internal validation, and external validation cohorts. Univariate and multivariate Cox proportional hazards models were then applied for variable selection. Initially, each variable was assessed using the univariate model. Afterward, potential interactions between selected covariates were checked for multicollinearity using the variance inflation factor (VIF) test, prior to multivariate analysis. Multicollinearity was considered significant if the VIF was greater than 5 or the tolerance was below 0.2. The multivariate Cox proportional hazards model was then used to include all variables that were statistically significant in the univariate analysis, with backward elimination applied. Variables that remained significant in the multivariate model (log-rank test, P < 0.05) were selected to construct the mortality risk prediction model. The results were reported as hazard ratios (HRs) with 95% confidence intervals (CIs). We applied restricted cubic splines (RCS) to examine how the risk of mortality relates nonlinearly to seven continuous serum biochemical markers (TBil, DBil, ALT, AST, ALP, ALB, and AFP) and established four knots at the 5th, 25th, 75th, and 95th percentiles for each variable (S1 Table and S1 Fig). For five of these markers (TBil, DBil, ALT, AST, and ALB), significant threshold effects were observed in relation to the 6-year mortality risk for patients with advanced schistosomiasis post-discharge (P value for nonlinearity < 0.05). Based on the RCS analysis, these five markers were divided into 2–3 subgroups. Meanwhile, ALP and AFP were classified into normal and abnormal categories according to medical reference values.

## Results

### Characteristics of cohort

**Fig 1:**
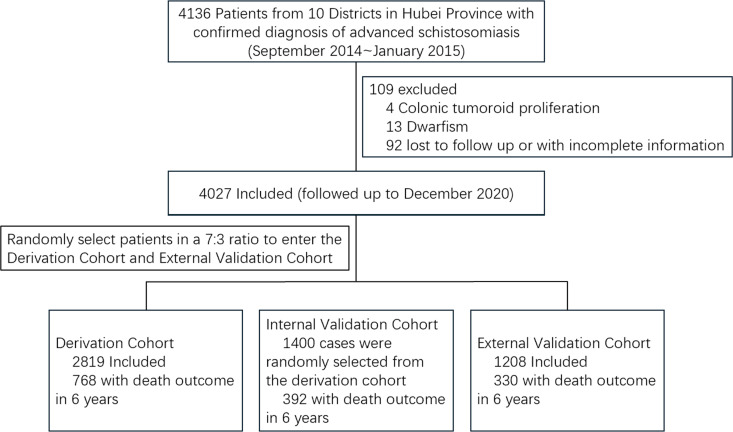
Flow chart of the study.

The study included 4,027 patients who were randomly assigned to derivation cohort and external validation cohort in a 7:3 ratio, with 2,819 and 1,208 patients in each cohort, respectively. All-cause mortality rate for both cohorts was approximately 27%. By the end of the follow-up period, 768 death events in the derivation cohort and 330 in the external validation cohort were recorded. Additionally, we randomly selected 1,400 patients from the derivation cohort for the internal validation cohort, which experienced 392 deaths, corresponding to a mortality rate of 28% (Fig 1).

The average age of patients in the cohort was approximately 61 years, with females comprising about 35%. In the derivation cohort, 43.8% of patients had a disease duration of over 5 years, compared to 42.1% in the internal validation cohort and 45.9% in the external validation cohort. In the derivation cohort, 16% of patients exhibited splenomegaly, and 84% presented with ascites, of whom 50.9% experienced ascites more than five times, and 40.3% had a history of splenectomy. Concurrent hepatitis B was found in 18.8% of patients in the derivation cohort and 17.6% in the validation cohort. Additionally, 47.1% of patients in the derivation cohort had comorbidities such as digestive system and cardiovascular diseases, while this percentage was 46.3% in the validation cohort. Laboratory data, including ALT, AST, DBil, TBil, and other indicators, were collected for all patients in both the derivation and validation cohorts, ensuring no statistical differences between the two cohorts. (Table 1).

**Table 1:** Baseline Characteristics of the Derivation, Internal, and External Validation Cohorts.

Variables		Cohort, No. (%) of patients			
		Derivation (n = 2819)	Internal validation (n = 1400)	External validation (n = 1208)	P value (Derivation vs External validation)
Demographics and clinical characteristics					
Age, mean (SD), years		61.56 (10.876)	61.59 (10.789)	61.48 (11.010)	0.828
Sex, women		990 (35.1%)	508 (36.3%)	423 (35.0%)	0.971
Course of disease, mean (SD), years	>5 years	1234 (43.8%)	590 (42.1%)	554 (45.9%)	0.226
	≤5 years	1585 (56.2%)	810 (57.9%)	654 (54.1%)	
Nourishment status	Well	387 (13.7%)	191 (13.6%)	150 (12.4%)	0.399
	General	2281 (80.9%)	1133 (80.9%)	985 (81.5%)	
	Poor	151 (5.4%)	76 (5.4%)	73 (6.0%)	
Clinical classification	Splenomegaly	452 (16.0%)	213 (15.2%)	176 (14.6%)	0.255
	Ascites	2367 (84.0%)	1187 (84.8%)	1032 (85.4%)	
Frequency of ascites	<5 times	1384 (49.1%)	698 (49.9%)	598 (49.5%)	0.837
	≥5 times	1435 (50.9%)	702 (50.1%)	610 (50.5%)	
History of splenectomy	No	1683 (59.7%)	838 (59.9%)	704 (58.3%)	0.401
	Yes	1136 (40.3%)	562 (40.1%)	504 (41.7%)	
Other disease	None	1490 (52.9%)	734 (52.4%)	649 (53.7%)	0.783
	Cardiovascular	345 (12.2%)	170 (12.1%)	134 (11.1%)	
	Digestive	603 (21.4%)	301 (21.5%)	260 (21.5%)	
	Other	381 (13.5%)	195 (13.9%)	165 (13.7%)	
Laboratory data					
TBil (μmol/L)	<18	1392 (49.4%)	698 (49.9%)	575 (47.6%)	0.302
	≥18	1427 (50.6%)	702 (50.1%)	633 (52.4%)	
DBil (μmol/L)	<6	1430 (50.7%)	719 (51.4%)	581 (48.1%)	0.13
	≥6	1389 (49.3%)	681 (48.6%)	627 (51.9%)	
ALT (u/L)	(15, 68)	2348 (83.3%)	1158 (82.7%)	1009 (83.5%)	0.853
	≥68	123 (4.4%)	64 (4.6%)	56 (4.6%)	
	≤15	348 (12.3%)	178 (12.7%)	143 (11.8%)	
AST (u/L)	<40	1418 (50.3%)	724 (51.7%)	594 (49.2%)	0.514
	≥40	1401 (49.7%)	676 (48.3%)	614 (50.8%)	
ALP (u/L)	Normal	2224 (78.9%)	1123 (80.2%)	973 (80.5%)	0.251
	Abnormal	595 (21.1%)	277 (19.8%)	235 (19.5%)	
ALB (g/L)	>40	1590 (56.4%)	775 (55.4%)	647 (53.6%)	0.097
	≤40	1229 (43.6%)	625 (44.6%)	561 (46.4%)	
HBsAg	Negative	2288 (81.2%)	1134 (81.0%)	995 (82.4%)	0.376
	Positive	531 (18.8%)	266 (19.0%)	213 (17.6%)	
AFP	Normal	2762 (98.0%)	1372 (98.0%)	1185 (98.1%)	0.902
	Abnormal	57 (2.0%)	28 (2.0%)	23 (1.9%)	

### Predicting all-cause mortality in the derivation cohort

**Fig 2:**
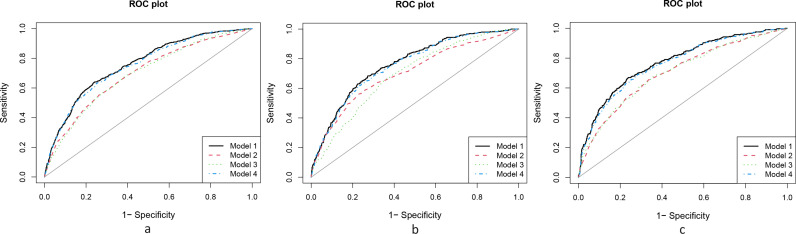
Model 1–4 Reclassification Performance Across Different Cohorts. Model 1 included Age, Frequency of ascites, Course of disease, ALT, AST, ALP, DBil, ALB, AFP and HBsAg. Model 2 included DBil, ALT, AST, ALB, HBsAg, ALP and AFP. Model 3 included Age, Frequency of ascites, Course of disease, HBsAg, AFP. Model 4 included Age, Course of disease, Frequency of ascites, ALT, AST, ALP, ALB and DBil. **a.** ROC Curves and AUC for the Derivation Cohort. Models 1 through 4 had AUCs of 0.759, 0.697, 0.693, and 0.751, respectively. **b.** ROC Curves and AUC for the Internal Validation Cohort. Models 1 through 4 had AUCs of 0.763, 0.703, 0.694, and 0.755, respectively. **c.** ROC Curves and AUC for the External Validation Cohort. Models 1 through 4 had AUCs of 0.774, 0.704, 0.704, and 0.763, respectively.

Sixteen variables, including age, sex, nutritional status, disease progression, types of complications, comorbidities, and various laboratory data, were collected for univariate and multivariate Cox proportional hazards regression analysis to evaluate their relationship with increased mortality risk. Our analysis identified ten variables associated with a higher risk of mortality: older age, disease duration exceeding 5 years, frequency of ascites exceeding 5 episodes, concurrent hepatitis B, and elevated baseline levels of ALT, AST, DBil, ALP, ALB, and AFP (Table 2). The ten-variable model (model 1) demonstrated the lowest Akaike Information Criterion (AIC) (2834.2) and the highest C statistic (0.759; 95% CI, 0.739-0.778). ROC curve analysis showed that the AUC value of model 1 was 0.759, surpassing those of models 2–4 (Fig 2). Simplified models incorporating only laboratory indicators DBil, ALT, AST, ALP, ALB, HBsAg, and AFP (model 2), or excluding DBil, ALT, AST, ALP, ALB (model 3), and excluding HBsAg, AFP (model 4), exhibited lower discrimination based on integrated discrimination improvement (IDI) and the C statistic. Model 3 had the lowest C statistic (0.693; 95% CI, 0.671-0.714). In terms of IDI, the performance of the reduced models showed minimal improvement (Table 3).

**Table 2:** Univariate and multivariate Cox proportional hazards regression analysis for variables selection.

Variables		Overall survival							
		Univariate analysis				Multivariate analysis			
		HR	95%CI		P	HR	95%CI		P
Age, years		1.049	1.042	1.057	<0.001	1.048	1.040	1.056	<0.001
Sex	Females/Males	0.875	0.752	1.018	0.083				NS
Nourishment status	Well	ref			0.348				NI
	General/	0.966	0.787	1.185	0.740				
	Poor/	1.199	0.853	1.684	0.296				
Course of disease, years	≤5 years/ > 5 years	1.215	1.052	1.404	0.008	1.548	1.319	1.816	<0.001
Clinical classification	Splenomegaly/Ascites	1.044	0.862	1.264	0.659				NI
Frequency of ascites	≥5 times/ < 5 times	1.323	1.147	1.526	<0.001	1.571	1.339	1.843	<0.001
History of splenectomy	None/Yes	1.376	1.185	1.598	<0.001				NS
Other disease	None	ref			0.170				NI
	Cardiovascular/	1.055	0.842	1.322	0.640				
	Digestive/	1.082	0.903	1.298	0.393				
	Other	1.261	1.028	1.547	0.026				
TBil (μmol/L)	≥18/ < 18	1.577	1.365	1.821	<0.001				NS
DBil (μmol/L)	≥6/ < 6	2.106	1.817	2.442	<0.001	1.881	1.604	2.205	<0.001
ALT (u/L)	(15, 68)	ref			<0.001	ref			<0.001
	≥68	1.740	1.311	2.309	<0.001	1.148	0.854	1.544	0.360
	≤15	1.200	0.976	1.475	0.083	1.580	1.261	1.980	<0.001
AST (u/L)	≥40/ < 40	1.660	1.437	1.918	<0.001	1.490	1.256	1.767	<0.001
ALP (u/L)	Abnormal/Normal	1.723	1.476	2.012	<0.001	1.239	1.051	1.460	0.011
ALB (g/L)	≤40/ > 40	2.333	2.018	2.698	<0.001	1.842	1.582	2.144	<0.001
HBsAg	Positive/Negative	1.328	1.122	1.573	0.001	1.443	1.212	1.717	<0.001
AFP	Abnormal/Normal	2.803	1.957	4.016	<0.001	2.142	1.481	3.098	<0.001

**Table 3:** Predictors of Mortality in Advanced Schistosomiasis and Model Performance in the Derivation Cohort.

Predictors		Models, Odds Ratio(95% CI)			
		Model 1. Age, Course of disease, Frequency of ascites, DBil, ALT, AST, ALP, ALB, HBsAg, AFP	Model 2. DBil, ALT, AST, ALP, ALB, HBsAg, AFP	Model 3. Age, Course of disease, Frequency of ascites, HBsAg, AFP	Model 4. Age, Course of disease, Frequency of ascites, DBil, ALT, AST, ALP, ALB
Age, mean (SD), years		1.0628 (1.0523-1.0734)		1.066 (1.056-1.076)	1.058 (1.048-1.069)
Course of disease, mean (SD), years	>5 years	1 [Reference]		1 [Reference]	1 [Reference]
	≤5 years	1.6545 (1.3484-2.03)		1.737 (1.426-2.115)	1.683 (1.374-2.062)
Frequency of ascites	<5 times	1 [Reference]		1 [Reference]	1 [Reference]
	≥5 times	1.7587 (1.4324-2.1594)		1.502 (1.236-1.824)	1.762 (1.437-2.161)
DBil (μmol/L)	<6	1 [Reference]	1 [Reference]		1 [Reference]
	≥6	2.1764 (1.7824-2.6576)	2.083 (1.723-2.518)		2.216 (1.817-2.704)
ALT (u/L)	(15, 68)	1 [Reference]	1 [Reference]		1 [Reference]
	≥68	1.4969 (0.9775-2.2923)	1.333 (0.886-2.006)		1.616 (1.063-2.458)
	≤15	1.7326 (1.2948-2.3185)	1.917 (1.449-2.537)		1.730 (1.294-2.312)
AST (u/L)	<40	1 [Reference]	1 [Reference]		1 [Reference]
	≥40	1.634 (1.317-2.0273)	1.449 (1.179-1.782)		1.655 (1.335-2.051)
ALP (u/L)	Normal	1 [Reference]	1 [Reference]		1 [Reference]
	Abnormal	1.3338 (1.0704-1.6619)	1.399 (1.134-1.725)		1.352 (1.087-1.682)
ALB (g/L)	>40	1 [Reference]	1 [Reference]		1 [Reference]
	≤40	2.1114 (1.7506-2.5465)	2.462 (2.057-2.945)		2.137 (1.774-2.574)
HBsAg	Negative	1 [Reference]	1 [Reference]	1 [Reference]	
	Positive	1.6621 (1.3151-2.1006)	1.297 (1.042-1.615)	1.746 (1.398-2.179)	
AFP	Normal	1 [Reference]	1 [Reference]	1 [Reference]	
	Abnormal	2.2384 (1.2533-3.9978)	2.161 (1.229-3.802)	3.220 (1.8506-1.851)	
Model Performance Measures					
AIC		2834.2	3038	3047.5	2859.1
C statistic		0.759 (0.739-0.778)	0.697 (0.676-0.719)	0.693 (0.671-0.714)	0.751 (0.731-0.771)
Difference		1 [Reference]^*^	0.0613 (0.0457-0.0770)	0.0659 (0.0491-0.0827)	0.00796 (0.00284-0.0131)
P value			<0.001	<0.001	0.0023
IDI (95%CI), %			0.0705 (0.0603-0.0806)	0.0782 (0.0672 - 0.0892)	0.0099 (0.0054 - 0.0144)
P value			<0.001	<0.001	<0.001

* Models 2–4 were evaluated against Model 1. In these comparisons, an increase in IDI values and positive differences in the C statistic greater than 0 suggest that the full model 1 performs better than the reduced models.

### Predicting all-cause mortality in the internal cohorts

In the internal validation cohort, the ten-variable model maintained excellent calibration and demonstrated a superior C statistic (0.763; 95% CI: 0.740 - 0.785) compared to models with fewer variables (Table 4). Additionally, evaluations using the IDI, categorical NRI and continuous NRI consistently favored the ten-variable model over the reduced-variable models (Table 4). Model 1, which includes ten variables, showed a notable enhancement in risk categories compared to model 2. Specifically, the continuous NRI increased significantly by 46.94% (95% CI, 35.63–58.25%). Compared to model 3, model 1 exhibited substantial improvement, with the continuous NRI increasing by 57.48% (95% CI, 45.45–69.51%) and the categorical NRI increasing by 20.1% (95% CI, 12.91–27.28%). Similarly, when compared to model 4, model 1 showed a moderate enhancement, with the continuous NRI increasing by 16.64% (95% CI, 6.72–26.56%) and the categorical NRI increasing by 4.34% (95% CI, 0.58–8.1%). Moreover, the IDI index indicated significant improvements in model 1 (all P values < 0.05) compared to models 2–4. The AUC value of model 1 (0.763) further underscores its superior predictive performance over model 2 in the internal cohort (Fig 2).

**Table 4:** Evaluating the Predictive Accuracy of Models for Mortality in Internal and External Validation Cohorts.

	Models, Measures of Predictive Performance							
	Model 1. Age, Course of disease, Frequency of ascites, DBil, ALT, AST, ALP, ALB, HBsAg, AFP		Model 2. DBil, ALT, AST, ALP, ALB, HBsAg, AFP		Model 3. Age, Course of disease, Frequency of ascites, HBsAg, AFP		Model 4. Age, Course of disease, Frequency of ascites, DBil, ALT, AST, ALP, ALB	
	Internal validation	External validation	Internal validation	External validation	Internal validation	External validation	Internal validation	External validation
Calibration intercept	-0.0422	-0.0414	-0.0409	-0.047	-0.0249	-0.0245	-0.0362	-0.0339
Calibration slope	0.9466	0.9453	0.9515	0.9431	0.9671	0.9692	0.9581	0.9569
B	0.1686	0.1615	0.1792	0.1793	0.1837	0.1771	0.171	0.1651
C statistic (95%CI)	0.763 (0.740 - 0.785)	0.774 (0.749 - 0.797)	0.703 (0.678 - 0.727)	0.704 (0.678 - 0.730)	0.694 (0.669 - 0.718)	0.704 (0.677 - 0.730)	0.755 (0.731 - 0.777)	0.763 (0.738 - 0.787)
Difference in C statistic (95%CI)^a^			0.0603 (0.0393 - 0.0814)	0.0693 (0.0436 - 0.0950)	0.0696 (0.0458 - 0.0933)	0.0698 (0.0443 - 0.0954)	0.00834 (0.000796 - 0.0159)	0.0106 (0.000758 - 0.0205)
P value			< 0.001	< 0.001	< 0.001	< 0.001	P = 0.0302	P = 0.0348
IDI (95%CI), %			0.0597 (0.0467-0.0727)	0.0894 (0.0718-0.1071)	0.0825 (0.0665-0.0985)	-0.09 (0.0722-0.1077)	0.0135 (0.006-0.0209)	0.0167 (0.007-0.0264)
P value			< 0.001	< 0.001	< 0.001	< 0.001	0.00037	0.00076
NRI (95%CI), %								
Continuous			0.4694 (0.3563-0.5825)	0.5748 (0.4545-0.6951)	0.6108 (0.4998-0.7218)	0.6001 (0.4783-0.722)	0.1664 (0.0672-0.2656)	0.1945 (0.0823-0.3068)
P value			< 0.001	< 0.001	< 0.001	< 0.001	0.00101	0.00068
Categorical^b^			0.1424 (0.0874-0.1975)	0.2671 (0.1967-0.3375)	0.201 (0.1291-0.2728)	0.2527 (0.1767-0.3287)	0.0434 (0.0058 -0.081)	0.0277 (0.0063-0.0616)
P value			< 0.001	< 0.001	< 0.001	< 0.001	0.02378	0.11072
Events, No. (%) ^c^			108 (27.55)	132 (40.00)	178 (45.41)	148 (44.85)	47 (11.99)	28 (8.48)
Nonevents, No. (%)			193 (19.15)	191 (21.75)	260 (25.79)	229 (26.08)	68 (6.75)	35 (3.99)
Overall, No. (%)			301 (21.50)	323 (26.74)	438 (31.29)	377 (31.21)	115 (8.21)	63 (5.22)

Abbreviations: IDI, integrated discrimination improvement; NRI, net reclassification improvement.

a Models 2–4 were evaluated against model 1. In these comparisons, an increase in IDI values and positive differences in the C statistic greater than 0 suggest that the full model 1 performs better than the reduced models.

b Risk categories include patients with < 10%, 10% ~ 30%, 30% or higher risk of death.

c NRI events: occurrence of death; nonevents: no occurrence of death.

### Predicting all-cause mortality in external validation cohorts

In the external validation cohort, Model 1, which included ten variables, demonstrated the highest C statistic (0.774; 95% CI: 0.749 - 0.797) in comparison to Models 2–4. The ten-variable model outperformed the reduced-variable models, as evidenced by favorable results in ROC curve analyses, IDI, and both continuous and categorical NRI analyses.

Although model 3 demonstrated better IDI performance (-0.09; 95% CI: 0.0722-0.1077) than model 1, the ten-variable model still retained higher predictive ability when considering the C statistic and NRI. Specifically, the ten-variable model showed a significant enhancement in risk classification accuracy compared to model 2 (categorical NRI: 26.71%; 95% CI: 19.67–33.75%), model 3 (continuous NRI: 25.27%; 95% CI: 17.67–32.87%), and model 4 (continuous NRI: 2.77%; 95% CI: 0.63–6.16%). Furthermore, model 1 exhibited improved IDI values (all P values < 0.05) compared to models 2–4 (Table 4).

### Clinical utility of the prediction model

**Fig 3:**
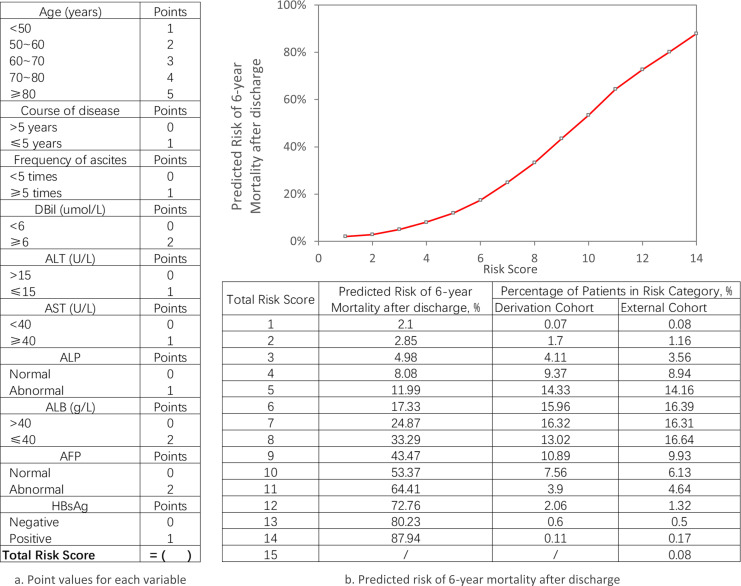
Risk Index for Post-Discharge Patients. **a.** Point values for each variable. To calculate a patient’s total risk score, sum the points assigned to each variable value, which will indicate the predicted 6-year mortality risk. **b.** Estimated 6-Year Mortality Risk After Discharge.

**Fig 4:**
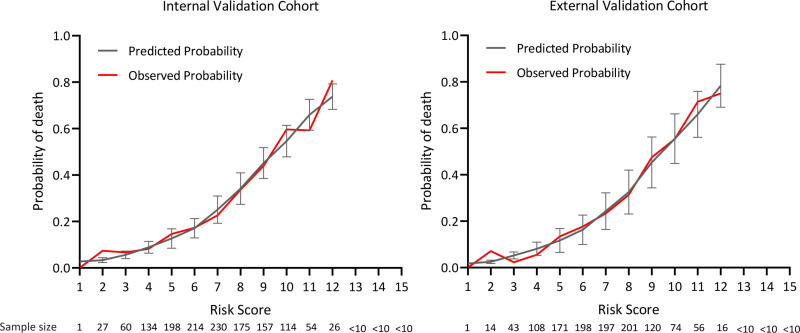
Comparison of Predicted and Observed 6-Year Mortality Risk Based on the Risk Index in Internal and External Validation Cohorts. Data are shown for the internal validation cohort (n = 1400) and the external validation cohort (n = 1208). Error bars reflect 95% confidence intervals. Risk scores ranged from 1 to 15 in both cohorts.

The practical implications of using the ten-variable risk index for managing follow-up care, across various predicted risk thresholds for advanced schistosomiasis, are illustrated in detail in Fig 3. Fig 4 presents a comparison between observed and predicted risks derived from the integer-based risk index across its entire range of internal and external cohorts (Fig 4).

## Discussion

We conducted analysis of an all-cause mortality predictive model in a cohort of 4,027 patients diagnosed with advanced schistosomiasis. Model 1, which incorporates ten variables—age, disease duration, frequency of ascites, DBil, ALT, AST, ALP, ALB, HbsAg, and AFP—demonstrated superior predictive performance in assessing the long-term mortality risk post-discharge over six-year period compared to reduced models. Through statistical indicators such as AUC, IDI, and NRI, we identified the model’s promising accuracy and efficiency. This model provided a convenient tool for predicting the prognosis of patients with advanced schistosomiasis, enabling more detailed, precise, and personalized management. It also enhanced understanding and awareness of advanced schistosomiasis, promoting a more rational allocation of medical resources.

In our previous study [21], we used seven variables (Age, AST, DBil, ALP, HBsAg, AFP, Clinical classification) to predicted 2-years mortality in 2210 patients with advanced schistosomiasis, which was helpful for decision-making of clinicians to some extent. Based on our current findings, we emphasized the extension to longer follow-up period and larger subject cohorts. Compared to previous studies, this research significantly expands the sample size and extends the follow-up period from two to six years. This expansion enhances the clinical relevance and applicability of our predictive models for predicting outcomes and management in advanced schistosomiasis. Additionally, we have refined the variables included in the model. Although the number of variables has increased, they are also straightforward and easily accessible. For instance, variables such as age, disease duration, and frequency of ascites can be easily obtained through standard medical history inquiry, while laboratory indicators can be quickly acquired through routine biochemical blood tests. Comparing to the previous study, we added three various (course of diseases, ALT and ALB) in model 1 and replace clinical classification (splenomegaly or ascites) with frequency of ascites based on univariate and multivariate cox proportional hazards regression analysis.

The course of the disease is an important indicator for assessing the risk of death in advanced schistosomiasis. Although preventive chemotherapy can reduce the incidence of acute schistosomiasis and chemotherapy such as praziquantel can kill adult schistosomes, regrettably, injury of eggs lay on liver, spleen, bladder and intestines cannot be blocked efficiently. Existing eggs in the body can continue to release toxins, triggering inflammatory and fibrotic reactions in the host, which may be the cause of intestinal inflammation, liver cirrhosis, splenomegaly or bladder cancer [25–28]. Data from the United Kingdom show that decompensation rates are 11% in the first year after cirrhosis diagnosis, and subsequently at a rate of 5–7% annually, with ascites being the predominant manifestation of decompensation [29]. Most patients with advanced schistosomiasis likely experience hepatic decompensation, and the course of decompensation is critical for clinical decision-making and prognosis assessment. Our study demonstrates a significant correlation between a disease duration of more than five years in advanced schistosomiasis and a six-year mortality risk.

There are four clinical subtypes of advanced schistosomiasis in China: ascites, megalosplenia, colonic tumoroid proliferation, and dwarfism. Ascitic type is a most common and serious complication of advanced schistosomiasis accounting for 65–90% and has a substantial effect on patients’ quality of life [23]. Ascites is a multifaceted indicator that can reflect patients’ nutritional status, infection and portal hypertension status [30]. Approximately 85% of ascites is caused by portal hypertension due to cirrhosis and the development of ascites signifies the onset of the decompensated phase of liver function [31]. The frequency of ascites is directly correlated with the incidence of liver-related complications and patient mortality [32]. Our study demonstrated that there is a significant association between the frequency of ascites and death events, indicating that ascites frequency greater than five times per year is a significant risk factor for mortality (Table 2). In fact, it is established that ascites frequency exceeding three times per year is classified as recurrent ascites, which is a precursor to refractory ascites, a condition that has a notably high mortality rate [33–35]. Therefore, compared to the mere occurrence of ascites, the frequency of ascites episodes may be more crucial for the prognostic assessment of advanced schistosomiasis and may hold greater clinical value. This is an important update of this model compared to our previous research.

Albumin contributes roughly 75% to the plasma oncotic pressure [36]. Liver is the main contributor to albumin recycling and circulating levels steadiness in physiological state [37]. Patients with advanced schistosomiasis have impaired albumin production and decreased blood albumin levels due to decompensated cirrhosis, which further leads to ascites production [38,39]. Furthermore, higher albumin level was negatively associated with the quality of life and disability weight [19]. Similar to frequency of ascites, ALB level is also important to longer-term survival in advanced schistosomiasis in our study (Table 2). This suggests that detecting and promptly correcting albumin levels are essential for patients with advanced schistosomiasis [40,41].

This study has several notable advantages. Firstly, patient data collected in this research is extremely valuable. Currently, international focus on advanced schistosomiasis is inadequate. The survival and prognosis of patients with advanced schistosomiasis are often conflated with those of general chronic schistosomiasis. However, advanced schistosomiasis has significantly higher mortality and disability rate compared to chronic schistosomiasis and should receive greater attention. The cohort in this study comprises over 4,000 patients with advanced schistosomiasis who have been followed for six years. This extensive sample size and prolonged follow-up duration provide exceptionally valuable data for the research of advanced schistosomiasis. Secondly, the clinical data collected in this study were both sufficient and comprehensive, covering basic information, course of disease, comorbidities and complications, as well as extensive laboratory and imaging data. This detailed data collection offered precise evidence for determining the inclusion and exclusion in our research and improved the model’s accuracy. Thirdly, the patients involved in this study exhibit high level of compliance, with a very low loss rate. China places considerable emphasis on patients with advanced schistosomiasis, providing independent registration and management, along with financial subsidies for treatment and follow-up. This policy ensures good compliance among the patients, resulting in the very low loss rate, with only 92 out of 4,136 patients lost to follow-up or having incomplete information. Finally, the model developed in this study was a convenient and efficient prognostic tool. The indicators involved in the model are commonly used in clinical practice, readily obtainable and cost-effective. Additionally, compared to our previous research and other machine learning studies, we have optimized the metrics by using the frequency of ascites instead of disease classification or ascites volume [21,23,42,43]. This approach is better suited for long-term prognostic analysis of advanced schistosomiasis and provide clinicians with an accessible evaluation tool that may help for clinical decision-making.

Our study also has some limitations. Firstly, due to the extremely low incidence of dwarfism and colonic tumoroid proliferation, the sample size for these conditions was too small, leading us to exclude them from the cohort. Therefore, the model developed in this study is primarily applicable to the prognostic analysis of patients with ascites and megalosplenia. Secondly, although we collected a comprehensive set of indicators and achieved good model performance, the candidate indicators did not include blood pressure, family care support, or detailed treatment regimens. Additionally, indicators for assessing end-stage liver function and complications, such as Prothrombin Activity (PTA), International Normalized Ratio (INR), serum creatinine levels and so on, were not included. Moreover, we were unable to collect serological markers of liver fibrosis such as Collagen IV (C IV), Procollagen III (PC III), Laminin (LN) and Hyaluronic acid (HA), as well as fibroscan and imaging data like ultrasound. Incorporating these indicators could potentially enhance the model’s predictive accuracy. Thirdly, since all patients in this study were from Hubei Province, additional research is needed to assess how well these findings apply to other countries and regions.

Based on the contribution of each variable to the model, we assigned scores to the variables and developed a scoring system (Fig 3) to assess the 6-year all-cause mortality risk for patients at varying levels. Our scoring system may help to overcome the limitations of the current processes in early intervention for complications of advanced schistosomiasis, personalized follow-up care, and the allocation of medical resources.

First, it provides an output of predictive tool that is understandable to patients and providers, which is helpful for individualized prognostic assessment and dynamic monitoring. All the variables in the scoring system are part of routine practice. Based on the data from regular follow-ups, the scoring system enables rapid prognostic assessment and dynamic comparisons, allowing for the identification of populations with more rapid disease progression. In addition, by dynamically comparing scores before and after intervention, the scoring system can help evaluate the effectiveness of the interventions.

Second, the scoring system may aid in patient risk stratification, thereby supporting personalized follow-up and optimizing treatment decisions. For example, when a patient’s score is 4 or below, the 6-year all-cause mortality rate is less than 10%; however, when the score exceeds 6, the 6-year mortality rate rises to over 20%. Risk assessment based on the scoring system helps adjust follow-up monitoring intervals, raises patients’ self-management awareness, and promotes the rational use of medical resources. This risk stratification can complement other scoring systems, assisting in the selection of the most appropriate treatment plan for patients at different risk levels and optimizing decisions in areas such as early medical interventions and the management of complications. For instance, it can serve as an adjunct to the Child-Pugh classification, helping assess whether patients with advanced schistosomiasis and esophageal or gastric varices need early transjugular intrahepatic portosystemic shunt (TIPS) surgery [44,45].

Thirdly, the scoring system may facilitate more targeted health education and healthcare resource allocation. Assuming a 10% and 30% threshold to categorize low, medium, and high-risk groups, the following approaches can be adopted: For patients with a score below 5, who fall into the low-risk category, the focus should be on emphasizing the importance of regular follow-up examinations and promoting a scientifically balanced diet and nutritional structure. For patients with a score between 5 and 7, classified as medium-risk, more stringent dietary guidance and disease monitoring should be provided. This includes avoiding hard or irritating foods, educating them on recording urine output and body weight, and raising awareness about observing and identifying signs of gastrointestinal bleeding. A study in China revealed that the pooled prevalence of depressive symptoms among patients with advanced schistosomiasis was 62.01%, which was particularly common among those with complications [46]. Psychological support should also be offered. For patients with a score of 8 or above, categorized as high-risk, health education for both patients and their families should prioritize emergency response measures for sudden severe conditions, such as massive hematemesis or confusion. Additionally, this scoring system can serve as an auxiliary tool for assessing treatment priorities, such as liver transplantation.

In the future, we will conduct further research in the following areas: (1) developing a predictive model for the progression of cirrhosis in advanced schistosomiasis patients, with the goal of guiding liver transplantation priority assessments; (2) creating a refined model for advanced schistosomiasis patients with comorbidities (such as co-infection with hepatitis B/C), to assess the impact of dual infections on prognosis.

To summarize, we have confirmed the indicators related to 6-years death events for patients with advanced schistosomiasis and constructed a predictive model for 6-year all-cause mortality. The model’s good predictive ability was demonstrated through internal validation and external validation. Using this model may assist doctors in making clinical decisions and developing more personalized follow-up plans for patients with advanced schistosomiasis, thereby improving their survival.

HR, hazard ratio; CI, confidence interval; NS, not significant; NI, not included.

## Supporting information

S1 Fig Restricted cubic splines (RCS) curves were used to examine the nonlinear relationships between the selected indices from the univariate analysis and the risk of mortality over a 6-year period. (TIF)

S1 Table The nonlinear effects test between continuous independent variables and 6-year mortality risk of patients with advanced schistosomiasis. (DOCX)

S1 Data Complete raw data underlying the analytical results. (CSV)

## References

[1] Gray DJ, Ross AG, Li Y-S, McManus DP. Diagnosis and management of schistosomiasis. BMJ. 2011;342:d2651. doi: 10.1136/bmj.d2651 21586478 PMC3230106

[2] McManus DP, Dunne DW, Sacko M, Utzinger J, Vennervald BJ, Zhou X-N. Schistosomiasis. Nat Rev Dis Primers. 2018;4(1):13. doi: 10.1038/s41572-018-0013-8 30093684

[3] Rinaldo D, Perez-Saez J, Vounatsou P, Utzinger J, Arcand J-L. The economic impact of schistosomiasis. Infect Dis Poverty. 2021;10(1):134. doi: 10.1186/s40249-021-00919-z 34895355 PMC8667389

[4] Colley DG, Bustinduy AL, Secor WE, King CH. Human schistosomiasis. Lancet. 2014;383(9936):2253–64. doi: 10.1016/S0140-6736(13)61949-2 24698483 PMC4672382

[5] Global incidence, prevalence, years lived with disability (YLDs), disability-adjusted life-years (DALYs), and healthy life expectancy (HALE) for 371 diseases and injuries in 204 countries and territories and 811 subnational locations, 1990-2021: a systematic analysis for the Global Burden of Disease Study 2021. Lancet. 2024;403(10440):2133-–2161. 10.1016/S0140-6736(24)00757-8 38642570 PMC11122111

[6] GBD 2016 DALYs and HALE Collaborators. Global, regional, and national disability-adjusted life-years (DALYs) for 333 diseases and injuries and healthy life expectancy (HALE) for 195 countries and territories, 1990-2016: a systematic analysis for the Global Burden of Disease Study 2016. Lancet. 2017;390(10100):1260–344. doi: 10.1016/S0140-6736(17)32130-X 28919118 PMC5605707

[7] Mutapi F, Maizels R, Fenwick A, Woolhouse M. Human schistosomiasis in the post mass drug administration era. Lancet Infect Dis. 2017;17(2):e42–8. doi: 10.1016/S1473-3099(16)30475-3 27988094 PMC7614913

[8] Zhang Z, Jiang Q. Schistosomiasis elimination. Lancet Infect Dis. 2011;11(5):345; author reply 346-7. doi: 10.1016/S1473-3099(11)70109-8 21530891

[9] Organization WH. Ending the neglect to attain the sustainable development goals: a global strategy on water, sanitation and hygiene to combat neglected tropical diseases, 2021–2030. Ending the neglect to attain the sustainable development goals: a global strategy on water, sanitation and hygiene to combat neglected tropical diseases, 2021–2030. 2021.

[10] Steinmann P, Keiser J, Bos R, Tanner M, Utzinger J. Schistosomiasis and water resources development: systematic review, meta-analysis, and estimates of people at risk. Lancet Infect Dis. 2006;6(7):411–25. doi: 10.1016/S1473-3099(06)70521-7 16790382

[11] Li S-Z, Zheng H, Abe EM, Yang K, Bergquist R, Qian Y-J, et al. Reduction patterns of acute schistosomiasis in the People’s Republic of China. PLoS Negl Trop Dis. 2014;8(5):e2849. doi: 10.1371/journal.pntd.0002849 24810958 PMC4014431

[12] Zongo D, Tiendrebeogo JMA, Ouedraogo WM, Bagayan M, Ouedraogo SH, Bougouma C, et al. Epidemiological situation of schistosomiasis in 16 districts of Burkina Faso after two decades of mass treatment. PLoS Negl Trop Dis. 2025;19(2):e0012858. doi: 10.1371/journal.pntd.0012858 39913556 PMC11813138

[13] Organization WH. WHO guideline on control and elimination of human schistosomiasis: World Health Organization. 2022. 35235279

[14] Lo NC, Bezerra FSM, Colley DG, Fleming FM, Homeida M, Kabatereine N, et al. Review of 2022 WHO guidelines on the control and elimination of schistosomiasis. Lancet Infect Dis. 2022;22(11):e327–35. doi: 10.1016/S1473-3099(22)00221-3 35594896

[15] Zhang L, He J, Yang F, Dang H, Li Y, Guo S, et al. Progress of schistosomiasis control in People’s Republic of China in 2023. Zhongguo Xue Xi Chong Bing Fang Zhi Za Zhi. 2024;36(3):221–7. doi: 10.16250/j.32.1374.2024116 38952305

[16] Zhang L, He J, Yang F, Dang H, Li Y, Guo S, et al. Progress of schistosomiasis control in People’s Republic of China in 2022. Zhongguo Xue Xi Chong Bing Fang Zhi Za Zhi. 2023;35(3):217–24. doi: 10.16250/j.32.1374.2023073 37455091

[17] Zhang LJ, Xu ZM, Yang F, He JY, Dang H, Li YL, et al. Progress of schistosomiasis control in People’s Republic of China in 2021. Zhongguo Xue Xi Chong Bing Fang Zhi Za Zhi. 2022;34(4):329–36. doi: 10.16250/j.32.1374.2022132 36116921

[18] Shen Z, Luo H. The impact of schistosomiasis on the Global Disease Burden: a systematic analysis based on the 2021 Global Burden of Disease study. Parasite. 2025;32:12. doi: 10.1051/parasite/2025005 39981999 PMC11843987

[19] Jia T-W, Utzinger J, Deng Y, Yang K, Li Y-Y, Zhu J-H, et al. Quantifying quality of life and disability of patients with advanced schistosomiasis japonica. PLoS Negl Trop Dis. 2011;5(2):e966. doi: 10.1371/journal.pntd.0000966 21358814 PMC3039691

[20] Li G, Lian L, Huang S, Miao J, Cao H, Zuo C, et al. Nomograms to predict 2-year overall survival and advanced schistosomiasis-specific survival after discharge: a competing risk analysis. J Transl Med. 2020;18(1):187. doi: 10.1186/s12967-020-02353-5 32375846 PMC7201698

[21] Li G, Huang S, Lian L, Song X, Sun W, Miao J, et al. Derivation and external validation of a model to predict 2-year mortality risk of patients with advanced schistosomiasis after discharge. EBioMedicine. 2019;47:309–18. doi: 10.1016/j.ebiom.2019.08.028 31451437 PMC6796502

[22] Li G, Zhou X, Liu J, Chen Y, Zhang H, Chen Y, et al. Comparison of three data mining models for prediction of advanced schistosomiasis prognosis in the Hubei province. PLoS Negl Trop Dis. 2018;12(2):e0006262. doi: 10.1371/journal.pntd.0006262 29447165 PMC5831639

[23] Jiang H, Deng W, Zhou J, Ren G, Cai X, Li S, et al. Machine learning algorithms to predict the 1 year unfavourable prognosis for advanced schistosomiasis. Int J Parasitol. 2021;51(11):959–65. doi: 10.1016/j.ijpara.2021.03.004 33891933

[24] Collins GS, Reitsma JB, Altman DG, Moons KGM. Transparent reporting of a multivariable prediction model for individual prognosis or diagnosis (TRIPOD): the TRIPOD statement. BMJ. 2015;350:g7594. doi: 10.1136/bmj.g7594 25569120

[25] Gryseels B, Polman K, Clerinx J, Kestens L. Human schistosomiasis. Lancet. 2006;368(9541):1106–18. doi: 10.1016/S0140-6736(06)69440-3 16997665

[26] Abdel Aziz N, Musaigwa F, Mosala P, Berkiks I, Brombacher F. Type 2 immunity: a two-edged sword in schistosomiasis immunopathology. Trends Immunol. 2022;43(8):657–73. doi: 10.1016/j.it.2022.06.005 35835714

[27] Chuah C, Jones MK, Burke ML, McManus DP, Gobert GN. Cellular and chemokine-mediated regulation in schistosome-induced hepatic pathology. Trends Parasitol. 2014;30(3):141–50. doi: 10.1016/j.pt.2013.12.009 24433721

[28] Stadecker MJ, Asahi H, Finger E, Hernandez HJ, Rutitzky LI, Sun J. The immunobiology of Th1 polarization in high-pathology schistosomiasis. Immunol Rev. 2004;201:168–79. doi: 10.1111/j.0105-2896.2004.00197.x 15361240

[29] Fleming KM, Aithal GP, Card TR, West J. The rate of decompensation and clinical progression of disease in people with cirrhosis: a cohort study. Aliment Pharmacol Ther. 2010;32(11–12):1343–50. doi: 10.1111/j.1365-2036.2010.04473.x 21050236

[30] Zou J, Li H, Deng G, Wang X, Zheng X, Chen J, et al. A novel prognostic nomogram for older patients with acute-on-chronic liver diseases (AoCLD): a nationwide, multicentre, prospective cohort study. Age Ageing. 2023;52(1):afac313. doi: 10.1093/ageing/afac313 36626326 PMC9831261

[31] Wong F. Management of refractory ascites. Clin Mol Hepatol. 2023;29(1):16–32. doi: 10.3350/cmh.2022.0104 35676862 PMC9845666

[32] Parvataneni S, Sarkis Y, Haugh M, Baker B, Tang Q, Nephew LD, et al. A comprehensive evaluation of emergency department utilization by patients with cirrhosis. Am J Gastroenterol. 2024;119(12):2444–54. doi: 10.14309/ajg.0000000000002905 38912688 PMC11617279

[33] Salerno F, Guevara M, Bernardi M, Moreau R, Wong F, Angeli P, et al. Refractory ascites: pathogenesis, definition and therapy of a severe complication in patients with cirrhosis. Liver Int. 2010;30(7):937–47. doi: 10.1111/j.1478-3231.2010.02272.x 20492521

[34] Jepsen P, Watson H, Macdonald S, Vilstrup H, Jalan R. MELD remains the best predictor of mortality in outpatients with cirrhosis and severe ascites. Aliment Pharmacol Ther. 2020;52(3):492–9. doi: 10.1111/apt.15882 32573818

[35] Garbuzenko DV, Arefyev NO. Current approaches to the management of patients with cirrhotic ascites. World J Gastroenterol. 2019;25(28):3738–52. doi: 10.3748/wjg.v25.i28.3738 31391769 PMC6676543

[36] Fernández J, Clària J, Amorós A, Aguilar F, Castro M, Casulleras M, et al. Effects of albumin treatment on systemic and portal hemodynamics and systemic inflammation in patients with decompensated cirrhosis. Gastroenterology. 2019;157(1):149–62. doi: 10.1053/j.gastro.2019.03.021 30905652

[37] Pyzik M, Rath T, Kuo TT, Win S, Baker K, Hubbard JJ, et al. Hepatic FcRn regulates albumin homeostasis and susceptibility to liver injury. Proc Natl Acad Sci U S A. 2017;114(14):E2862–71. doi: 10.1073/pnas.1618291114 28330995 PMC5389309

[38] Bernardi M, Angeli P, Claria J, Moreau R, Gines P, Jalan R, et al. Albumin in decompensated cirrhosis: new concepts and perspectives. Gut. 2020;69(6):1127–38. doi: 10.1136/gutjnl-2019-318843 32102926 PMC7282556

[39] Arroyo V, García-Martinez R, Salvatella X. Human serum albumin, systemic inflammation, and cirrhosis. J Hepatol. 2014;61(2):396–407. doi: 10.1016/j.jhep.2014.04.012 24751830

[40] Sort P, Navasa M, Arroyo V, Aldeguer X, Planas R, Ruiz-del-Arbol L, et al. Effect of intravenous albumin on renal impairment and mortality in patients with cirrhosis and spontaneous bacterial peritonitis. N Engl J Med. 1999;341(6):403–9. doi: 10.1056/NEJM199908053410603 10432325

[41] Caraceni P, O’Brien A, Gines P. Long-term albumin treatment in patients with cirrhosis and ascites. J Hepatol. 2022;76(6):1306–17. doi: 10.1016/j.jhep.2022.03.005 35589252

[42] Hong Z, Zhang S, Li L, Li Y, Liu T, Guo S, et al. A nomogram for predicting prognosis of advanced schistosomiasis japonica in dongzhi county-a case study. Trop Med Infect Dis. 2023;8(1):33. doi: 10.3390/tropicalmed8010033 36668940 PMC9866143

[43] Liu X-F, Li Y, Ju S, Zhou Y-L, Qiang J-W. Network analysis and nomogram in the novel classification and prognosis prediction of advanced schistosomiasis Japonica. Am J Trop Med Hyg. 2023;108(3):569–77. doi: 10.4269/ajtmh.22-0267 36689944 PMC9978554

[44] Nicoară-Farcău O, Han G, Rudler M, Angrisani D, Monescillo A, Torres F, et al. Effects of early placement of transjugular portosystemic shunts in patients with high-risk acute variceal bleeding: a meta-analysis of individual patient data. Gastroenterology. 2021;160(1):193-205.e10. doi: 10.1053/j.gastro.2020.09.026 32980344

[45] Stanley AJ, Laine L. Management of acute upper gastrointestinal bleeding. BMJ. 2019;364:l536. doi: 10.1136/bmj.l536 30910853

[46] Qi Y-X, Huang M-R, Sun H-Y, Wu X-Y, Liu Z-T, Lu D-B. Prevalence of depressive symptoms in patients with advanced schistosomiasis in China: a systematic review and meta-analysis. PLoS Negl Trop Dis. 2024;18(3):e0012003. doi: 10.1371/journal.pntd.0012003 38452104 PMC10950241

