# Supplementary figures and images for: A model predicting the 6-year all cause mortality of patients with advanced schistosomiasis after discharge: Derived from a large population-based cohort study
Source: PLoS Negl Trop Dis. 2025 May 27;19(5):e0013134. doi: 10.1371/journal.pntd.0013134 (PMC12136624; doi:10.1371/journal.pntd.0013134)

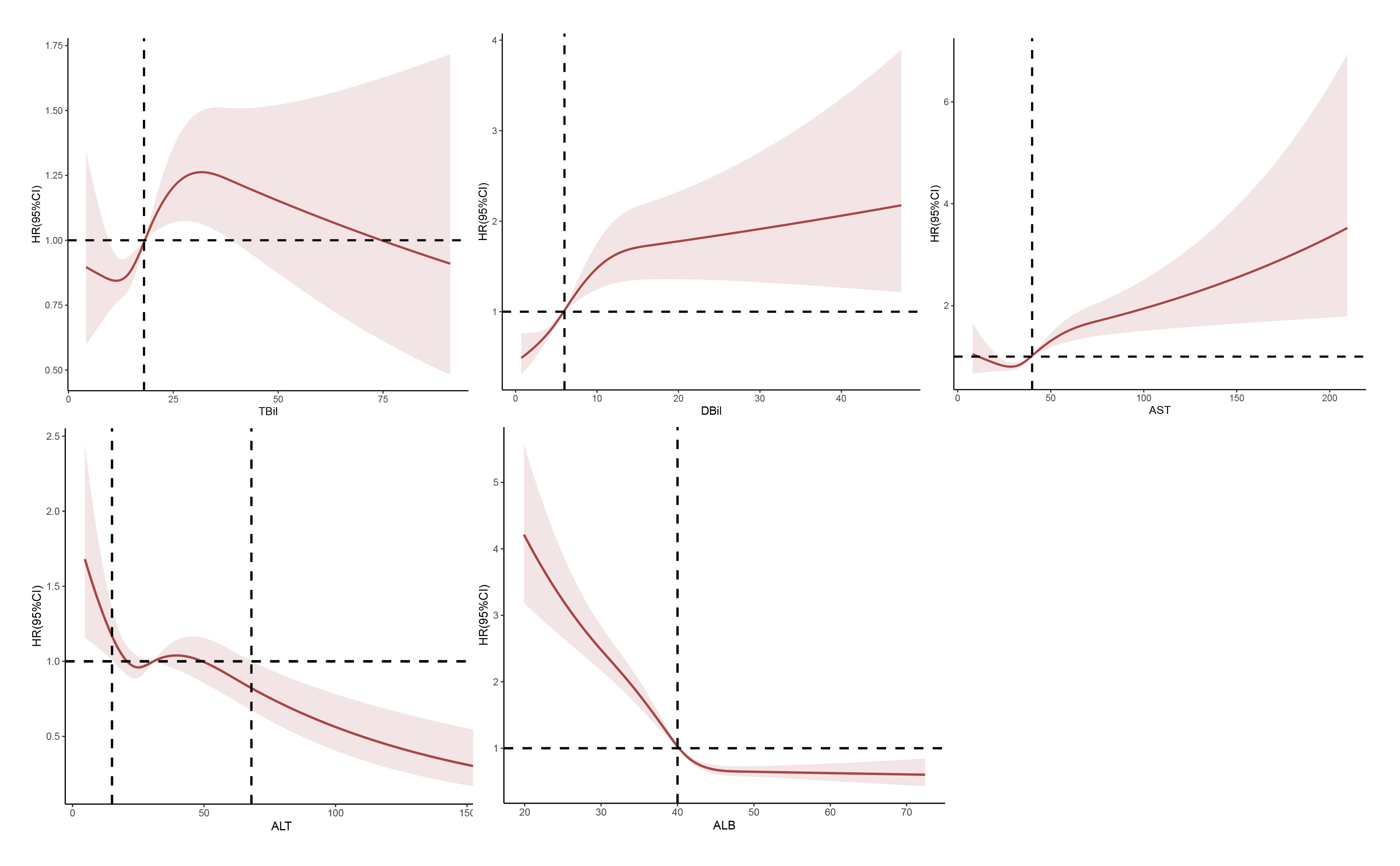

Supplement: S1 Fig — (TIF) [file pntd.0013134.s001.tif]
