# Supplementary material for: A model predicting the 6-year all cause mortality of patients with advanced schistosomiasis after discharge: Derived from a large population-based cohort study
Source: PLoS Negl Trop Dis. 2025 May 27;19(5):e0013134. doi: 10.1371/journal.pntd.0013134 (PMC12136624; doi:10.1371/journal.pntd.0013134)
Supplement: S1 Table — (DOCX) [file pntd.0013134.s002.docx]

**Table S1.** The nonlinear effects test between continuous independent variables and 6-year mortality risk of patients with advanced schistosomiasis

| Variables | Chi-Square | df | P value for Nonlinear |
| --- | --- | --- | --- |
| TBil | 13.9 | 2 | 0.0010 |
| DBil | 37.94 | 2 | <.0001 |
| AST | 13.14 | 2 | 0.0014 |
| ALT | 7.3 | 2 | 0.0254 |
| ALP | 3.71 | 2 | 0.1566 |
| ALB | 78.58 | 2 | <.0001 |
